# Precision and Normative Values of a New Computerized Chart for Contrast Sensitivity Testing

**DOI:** 10.1038/s41598-019-52987-9

**Published:** 2019-11-12

**Authors:** Giacomo Savini, Antonio Calossi, Domenico Schiano-Lomoriello, Piero Barboni

**Affiliations:** 1 IRCCS - Fondazione Bietti, Rome, Italy; 2 Department of Physics (Optics and Optometry), University of Florence, Florence, Italy; 3 Studio Oculistico d’Azeglio, Bologna, Italy; 4 Scientific Institute San Raffaele, Milan, Italy

**Keywords:** Eye manifestations, Imaging and sensing

## Abstract

The purpose was to define a normative database for a grating test for contrast sensitivity, based on a chart monitor with high-definition liquid crystal display, and validate its measurements by assessing their repeatability and determining responsiveness to cataract surgery. Three samples were analyzed: (1) healthy volunteers to assess the repeatability of measurements, (2) healthy subjects to develop the normative database, (3) patients undergoing cataract surgery. All subjects were tested with the grating contrast sensitivity test (Vision Chart, CSO) at 1.5, 3, 6, 12 and 18 cycles per degree. The instrument software progressively reduces the contrast of the gratings according to the Quick Estimate by Sequential Testing (QUEST) procedure. In the subjects of the first sample, three consecutive measurements were taken and repeatability was assessed on the basis of the intra-session test-retest variability and the coefficient of variation. The test offered high repeatability, with test-retest variability ranging between 0.05 and 0.23 Log CS and the coefficient of variation between 0.61 and 4.21%. Normative data did not show a normal distribution. The highest median values were observed at 1.5, 3 and 6 cycles per degree frequencies. At these frequencies a ceiling effect was evident. In cataract patients, postoperative values showed an improvement at all spatial frequencies. In conclusion, the new contrast sensitivity test provides repeatable measurements that can be used for clinical purposes. In patients with healthy eyes and good vision, attention has to be paid to the ceiling effect.

## Introduction

Over the last thirty years, measurement of contrast sensitivity (CS) has gained increasing relevance in ophthalmology, since it can provide useful information about a patient’s visual function that may not be revealed by visual acuity. CS has been used to investigate the clinical outcomes of corneal refractive surgery and intraocular lens (IOL) implantation^1–3^; it has also been found to be reduced in patients with glaucoma^4,5^, age-related macular degeneration^6^, neurological diseases^7,8^, and other pathological conditions^9^.

Different methods to assess CS have been described. They can be classified into optotype tests and grating tests. The former, which investigate only one spatial frequency by using optotypes with reduced contrast, include, for example, the Pelli-Robson chart (Haag-Streit UK, Essex, UK)^10^, the Mars Letter CS test (Mars Perceptrix, Chappaqua, NY)^11^, and the Rabin small letter contrast test (Precision Vision, Woodstock, IL)^12^. Grating tests, by contrast, investigate different spatial frequencies and were developed after the CS vision test chart originally introduced by Ginsburg in 1984^13^. A plot of CS over a range of spatial frequencies gives the contrast sensitivity function (CSF). A log scale of CS is normally used, because psychophysical measurements are logarithmic in nature. Grating tests typically contain circular plates of sine wave gratings that are either vertical or tilted 15° to the right or left. There are many commercially available CSF grating tests, such as the Vision Contrast Test System (VCTS) 6000 and 6500 (Vistech Consultants Inc., Dayton, OH) and the Functional Acuity Contrast Test (FACT, Vistech Consultants Inc.), two wall-mounted charts requiring external illumination, and the CSV-1000 (Vector Vision), which is retroilluminated. These tests have been widely used for almost thirty years, but may soon be replaced by similar examinations based on modern computer technology. Examples of computer-based assessments include the Freiburg Visual Acuity and Contrast Test (FrACT)^14^, the Spaeth/Richman Contrast Sensitivity (SPARCS)^15^, and the Quick CSF method^16^, which can be installed on a tablet^17^. Another example is the CS test included in the Vision Chart (CSO, Florence, Italy), a computerized optotype chart monitor with a 19-inch high-definition liquid crystal display (LCD). This instrument, which can measure CS by means of a grating test with five spatial frequencies, has two advantages over traditional charts: 1) its luminance is constant and depends on the monitor setting and 2) is part of a computer that can perform additional vision tests (e.g., visual acuity, refraction, binocular vision, and color tests). Like any computer-based test, it addresses the shortcomings of chart tests such as fading prints. On the other hand, it suffers from the limitations of LCDs, mainly represented by the lack of low levels of contrast ratios^18^. The CS grating test measurements provided by the Vision Chart have not yet been investigated (only the Pelli-Robson test performed with this instrument has been analyzed and found to offer repeatable measurements)^19^. Therefore, the aims of this study were to define a normative database of these measurements and to validate them by assessing their repeatability and determining their responsiveness to cataract surgery.

## Methods

### Subjects

Three samples were analyzed: the first included healthy volunteers from the staff of GB Bietti Foundation and was used to assess the repeatability of measurements, the second included healthy subjects examined for minor refractive defects and was used to develop the normative database, and the third included a series of consecutive patients before and after cataract surgery. Patients undergoing cataract surgery were enrolled on condition that their postoperative corrected distance visual acuity was 20/20 or better. All subjects received a standard ophthalmic clinical evaluation consisting of a slit-lamp and fundus examination, visual acuity testing, and intraocular pressure measurement.

Healthy subjects (samples 1 and 2) were enrolled on condition that their corrected distance visual acuity was 20/20 or better, the spherical equivalent of their refraction was within 3 diopters (D), they had not received any kind of ocular surgery, they had not any ocular disease, and their lens was fully transparent. The study design and protocol were approved by the Institutional Ethics Committee of GB Bietti Foundation and adhered to the tenets of the Declaration of Helsinki. Subjects gave informed written consent before participation.

### Contrast sensitivity test

All subjects were tested with the grating CS test (software version 2.2) included in the Vision Chart (CSO) at a distance of 4 meters; measurements were made monocularly with optimal refractive correction and under natural pupil dilation. The unit allows CS measurement at 1.5, 3, 6, 12 and 18 cycles per degree (cpd). The grating stimuli were presented as Gabor patches. Sine wave gratings are displayed in circular plates that are either vertical or tilted 15° to the right or left. Each sine wave grating appears as an evenly illuminated background (125 cd/m^2^) with an amplitude of 5.4° × 4.3° and includes a central area with full contrast (3.9°) and a peripheral annulus with blending contrast. A half-Gaussian ramp was added to each stimulus to minimize edge effects. For each spatial frequency, the instrument software progressively reduces the contrast of gratings according to the Quick Estimate by Sequential Testing (QUEST) procedure, with no fixed steps^20^. The initial stimulus value is determined by the mode (maximum likelihood) of the experimenter’s a priori knowledge of the probability density function of threshold values over the population. The subject’s response is then used to construct a new probability density function using Bayes’s rule. The next stimulus is presented at the new most likely threshold. The device can produce a range of contrast between 0.02 and 2.25 logarithms of CS (LogCS, Michelson’s contrast), corresponding to a percentage ranging between 0.56 and 96.5%, with a minimum step of 0.01 LogCS. When all spatial frequencies have been tested, the instrument software provides a chart with a CSF measurement expressed as the LogCS or contrast percent.

### Testing procedure

With the window shaded, the room was illuminated by neon lamps, positioned so as to cause minimal glare. The room illumination at the level of the subject’s chair was 200 lux. All subjects were instructed to indicate the orientation of each grating, even when they were no longer able to detect them (3-alternative forced choice, AFC). The examination was always carried out with distance-corrected manifest refraction.

In healthy subjects (samples 1 and 2), one eye was randomly selected for the study. In the subjects of sample 1, three consecutive measurements were taken to test the repeatability. In patients with cataract (sample 3), the responsiveness of the CS measurements to uncomplicated cataract surgery was evaluated by testing patients once before and once one month after surgery. The test was administered for this group in the same manner as described earlier. All cataract patients underwent small-incision phacoemulsification in the study eye, with implantation of a monofocal posterior chamber intraocular lens (Acrysof SN60WF, Alcon, Ft. Worth, TX) by the same surgeon.

### Data analysis

In the present study, the term repeatability was used according to the definition of the International Organization for Standardization^21^, which considers it a part of accuracy. Accuracy includes trueness and precision. Trueness is the inverse of bias and is obtained by comparing the measurement result with the accepted reference (conventional true) value. Precision is the inverse of statistical uncertainty and is normally expressed in terms of standard deviation (SD). The factors involved include (1) the operator, (2) the equipment used, (3) the equipment calibration, (4) the environment, and (5) the elapsed time between measurements. Precision can be assessed by means of repeatability and reproducibility. Under repeatability conditions, measurements are repeated without changing factors 1 to 5, which therefore do not contribute to the variability of the measurement result. Under reproducibility conditions, those factors can vary (for example measurements are taken by two operators or at different times).

Repeatability was assessed on the basis of the intra-session test-retest variability and the coefficient of variation (COV). These are defined as follows:

1. Test-retest variability (also known as repeatability or limits of repeatability). This is calculated by multiplying the pooled within-subject SD (s_w_) by 2.77^22^. On the basis of repeatability, it can be expected that the difference between 2 measurements for the same subject will be less than 2.77 s_w_ for 95% of pairs of observations.
2. Coefficient of variation (COV). This is calculated as the s_w_ divided by the mean of the measurements and expressed as a percentage^23^.

For comparative purposes, we also considered the Coefficient of Repeatability (COR), calculated by multiplying the pooled within-subject SD (s_w_) by 1.96, as this value has been used by several investigators.

### Statistics

All statistical analyses were performed by means of Instat (version 3.10, GraphPad, La Jolla, CA). Normality of data distribution was assessed by means of the Kolmogorov-Smirnov test. Comparisons of CS values between healthy and cataract patients were carried out with the Mann-Whitney test, while a paired t-test was used to compare the preoperative and postoperative values.

Since no normative data for this test were available, we aimed to enroll at least 50 eyes. With this sample size and with the standard deviation we observed (0.25 LogCS with a frequency of 18 cpd), at a confidence level of 95%, we could estimate a precision level of 7%^24^.

## Results

The repeatability of CS measurements was tested in 24 healthy subjects (sample 1) with a mean age of 33.3 ± 9.1 years. The repeatability was high, although it decreased slightly at higher spatial frequencies, as the COV increased from 1.66% at 1.5 cpd to 4.21% at 18 cpd. Table 1 shows the test-retest variability and the COV for each spatial frequency.

**Table 1:** Repeatability of contrast sensitivity measurements. LogCS = logarithms of contrast sensitivity; cpd = cycles per degree.

	1.5 cpd	3 cpd	6 cpd	12 cpd	18 cpd
Test-retest variability (Log CS)	0.11	0.05	0.10	0.22	0.23
Coefficient of Repeatability (Log CS)	0.07	0.03	0.07	0.15	0.16
Coefficient of Variation (%)	1.66	0.61	1.55	3.87	4.21

**Figure 1:**
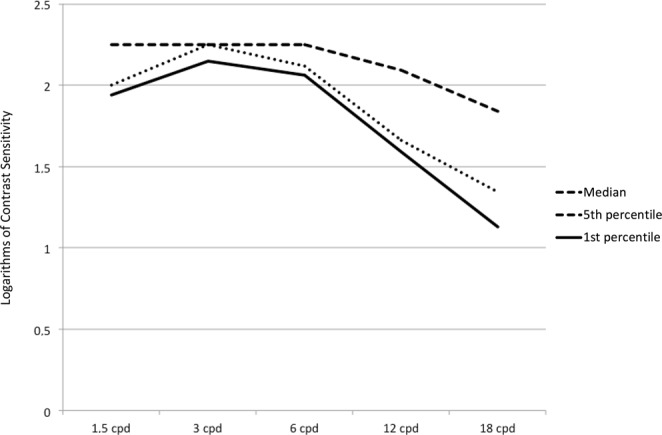
Distribution of CS measurements in the healthy sample.

Sample 2 included 57 healthy subjects (mean age = 39.8 ± 9.5 years; range: 20–50 years) who were tested to develop the normative database. Their descriptive statistics are shown in Table 2 and illustrated in Fig. 1.

**Table 2:** Descriptive statistics of contrast sensitivity measurements in healthy subjects. All values are reported as logarithms of contrast sensitivity. SD = standard deviation; cpd = cycles per degree.

	1.5 cpd	3 cpd	6 cpd	12 cpd	18 cpd
Median	2.25	2.25	2.25	2.09	1.84
95^th^ percentile	2.25	2.25	2.25	2.24	2.10
5^th^ percentile	2.00	2.25	2.12	1.66	1.34
1^st^ percentile	1.94	2.15	2.06	1.59	1.13

No measurements of spatial frequencies showed a normal distribution except for 18 cpd. The highest median values were observed at the 1.5, 3 and 6 cpd frequencies. At these frequencies a ceiling effect was evident, as most subjects (respectively, 54.4, 96.5, and 80.7%) scored the highest CS value possible.

Sample 3 included 15 patients (mean age: 78.9 ± 5.4 years) who underwent cataract surgery. In this group, CS values were normally distributed. Preoperative values were lower than those of healthy subjects (p < 0.0001 at all spatial frequencies). Table 3 reports the preoperative and postoperative values and shows an improvement at all spatial frequencies after surgery.

**Table 3:** Preoperative and postoperative logarithms of contrast sensitivity values in patients who underwent cataract surgery.

	1.5 cpd	3 cpd	6 cpd	12 cpd	18 cpd
Preop mean ± SD (interquartile range)	1.88 ± 0.14 (1.84–1.98)	1.92 ± 0.26 (1.71–2.08)	1.43 ± 0.43 (1.27–1.6)	0.79 ± 0.36 (0.66–0.97)	0.64 ± 0.35 (0.52–0.84)
Postop mean ± SD (interquartile range)	2.06 ± 0.11 (2.03–2.15)	2.14 ± 0.08 (2.11–2.25)	2.06 ± 0.32 (2.11–2.25)	1.64 ± 0.63 (1.52–1.93)	1.32 ± 0.66 (0.95–1.72)
P value	0.0277	0.0058	0.0001	0.0002	0.0134

## Discussion

The primary aim of this study was to provide readers with a normative database for CSF obtained with a new computerized chart. Since all subjects in the main sample were healthy, young and with minor refractive defects, the values reported in Table 2 can be considered a useful reference for both daily clinical routine and research. In addition, we found that measurements had good repeatability and could differentiate preoperative from postoperative vision in patients undergoing cataract surgery. These two latter observations confirm the validity of the test.

The results in the healthy subjects (Table 2 and Fig. 1) show that the highest CS values were obtained at the 3 and 6 cpd spatial frequencies, for which the 5^th^ percentile was, respectively, 2.25 and 2.12 LogCS. Pesudovs *et al*. reported similar outcomes with the FACT chart in healthy subjects. In the same study, the VCTS wall-chart test produced slightly different results, as the highest CS was found at 6 cpd^25^.

Figure 1 shows that the distribution of CS in healthy subjects, as measured by the computerized display tested in this study, is skewed to the highest values especially at the lowest frequencies, due to the large percentage of eyes that achieved the maximum possible score. Such a distribution has two opposite consequences: on the one hand it is expected to improve the sensitivity of this test in identifying diseases or conditions with even subtle CS loss, since the measured values of more than 95% of healthy cases lie in a very narrow interval and any deviation from this interval can be considered abnormal. On the other hand, it reveals a clear ceiling effect, which may mask differences between groups of patients when assessing CS at near-normal levels, such as after refractive surgery. The ceiling effect is a problem common to other CS tests, like the FACT chart^25,26^. With the computerized chart investigated in this study it is likely to depend on the LCD technology. Computer monitors are preferred over traditional CS tests with paper cards and charts because the latter reduce the range and resolution of contrast stimuli. Computerized CS tests are less affected with these issues and offer several advantages for research and clinical vision testing^27^. Historically, most psychophysical studies about CSF were conducted using cathode ray tube (CRT) displays, which allowed an excellent resolution of luminance through a combination of the colour outputs of graphic cards^28,29^. Nowadays, CRT displays are no more available and LCD displays took their place. Unfortunately, the grey-scale resolution of digital LCD monitors, based on 256 steps, is below the sensitivity of the human eye. Therefore, despite the use of dithering technology^30^, in the region of the higher CS values, the LCD chart suffers from ceiling effects.

Several studies have investigated the precision of CS tests, although in all cases they assessed only the reproducibility (and not repeatability). Moderate to poor reproducibility has been reported for measurements taken with one of the most commonly used methods, the VCTS wall-chart test^25,26,31–33^. The COR values reported by Elliott *et al*. (COR = 0.40 LogCS at the spatial frequency of 6cpd)^31^, Pesudovs *et al*. (CORs between 0.26 and 0.54 depending on the spatial frequencies)^25^ and Hong *et al*. (CORs between 0.09 and 0.29)^26^ are higher than the CORs observed in the present study and suggest a higher precision of the computerized chart (although a direct comparison is not totally correct, because we assessed intrasession repeatability and not intersession reproducibility). The poor precision of the VCTS wall-chart test can be explained by different causes. Some authors attribute it to the large step sizes (0.25 log units)^25,34^. On the other hand, Elliott *et al*. speculated that the poor reproducibility is due to the fact that this test relies on a non-forced-choice, criterion-dependent psychophysical technique: it allows subjects to make a “blank” response when they are not able to indicate the orientation of a grating and cannot see anything^31^. Previous studies reported that this strategy is less reliable than forced-choice ones^35,36^. In this context, the AFC approach may explain the good repeatability of the computerized chart tested in this study. For the same reason the FACT, which is based on AFC, showed lower CORs (i.e., better reproducibility) than the VCTS wall-chart test^25^.

The COR values of CS measurements in healthy subjects with the computerized chart were also lower than those obtained with the FACT chart^25,26^.

Responsiveness to cataract surgery is not a surprise. Previous studies using different methods found that CS significantly improves after cataract surgery^37^, and the computerized chart investigated by us led to the same conclusion. Improvement was higher at higher spatial frequencies, as expected, since with most cataracts contrast sensitivity decline is more likely to occur at intermediate and high spatial frequencies^38^.

This study has some limitations that warrant mention. First, we did not assess reproducibility, i.e. we did not perform measurements on the same subjects on different days. This is essential in order to confirm that this test can be used to detect longitudinal changes in CSF and will be the subject of a future study. Second, we did not correlate CS measurements of the computerized chart test to those provided by other instruments or to other tests investigating visual quality. Third, we did not measure CSF in healthy subjects older than 50, who represent the population where this test may be more frequently used (however, we specifically aimed to investigate young subjects in order to collect the highest values, which should represent the real normative database).

In conclusion, our data show that the new computerized chart offers a reliable test to measure CSF in healthy subjects and can be used for clinical and research purposes.
